# New treatment approaches for *Clostridioides difficile* infections: alternatives to antibiotics and fecal microbiota transplantation

**DOI:** 10.1080/19490976.2024.2337312

**Published:** 2024-04-09

**Authors:** Tomaž Bratkovič, Abida Zahirović, Maruša Bizjak, Maja Rupnik, Borut Štrukelj, Aleš Berlec

**Affiliations:** a Faculty of Pharmacy, University of Ljubljana, Ljubljana, Slovenia; b Department of Biotechnology, Jožef Stefan Institute, Ljubljana, Slovenia; c National Laboratory for Health, Environment and Food, Prvomajska 1, Maribor, Slovenia; d Faculty of Medicine, University of Maribor, Maribor, Slovenia

**Keywords:** *Clostridioides difficile*, treatment strategy, microbiota, bacteriophages, antibodies, immunomodulation

## Abstract

*Clostridioides difficile* causes a range of debilitating intestinal symptoms that may be fatal. It is particularly problematic as a hospital-acquired infection, causing significant costs to the health care system. Antibiotics, such as vancomycin and fidaxomicin, are still the drugs of choice for *C. difficile* infections, but their effectiveness is limited, and microbial interventions are emerging as a new treatment option. This paper focuses on alternative treatment approaches, which are currently in various stages of development and can be divided into four therapeutic strategies. Direct killing of *C. difficile* (i) includes beside established antibiotics, less studied bacteriophages, and their derivatives, such as endolysins and tailocins. Restoration of microbiota composition and function (ii) is achieved with fecal microbiota transplantation, which has recently been approved, with standardized defined microbial mixtures, and with probiotics, which have been administered with moderate success. Prevention of deleterious effects of antibiotics on microbiota is achieved with agents for the neutralization of antibiotics that act in the gut and are nearing regulatory approval. Neutralization of *C. difficile* toxins (iii) which are crucial virulence factors is achieved with antibodies/antibody fragments or alternative binding proteins. Of these, the monoclonal antibody bezlotoxumab is already in clinical use. Immunomodulation (iv) can help eliminate or prevent *C. difficile* infection by interfering with cytokine signaling. Small-molecule agents without bacteriolytic activity are usually selected by drug repurposing and can act via a variety of mechanisms. The multiple treatment options described in this article provide optimism for the future treatment of *C. difficile* infection.

## 1. Introduction

*Clostridioides difficile* is a Gram-positive strictly anaerobic, spore-forming bacterium that is found widely distributed in the environment, with the main habitat being the intestines of humans and various animals. Infection begins with the ingestion of spores that germinate in the small intestine and multiply in the colon. Colonization is favored by dysbiosis of the intestinal microbiota. *C. difficile* causes a spectrum of pathologic conditions ranging from mild self-limiting diarrhea, to serious diarrhea, pseudomembranous colitis, and life-threatening fulminant colitis that can lead to death.^1,2^ Recurrence occurs in nearly 20% of patients after initial *C. difficile* infection (CDI), and is one of the most important clinical problems.^1^ *C. difficile* causes approximately 780,000 infections and 49,000 deaths each year in the United States and Europe.^2^ In addition to mortality and reduced quality of life, the cost of treating and managing CDI is substantial ($800 million in the United States and €3,000 million in Europe annually).^3^

Two factors play an important role in intestinal pathogenesis: (i) the suppression of the resident intestinal microbiota by antibiotic administration and (ii) the production of exotoxins responsible for intestinal symptoms.^4^ Risk factors that also contribute to infection include advanced age, chemotherapy, use of proton pump inhibitors, chronic kidney disease, chronic liver disease, and malnutrition.^5^ Antibiotic use, especially in the hospital setting, is the major factor in the development of CDI because it causes disruption of the normal intestinal microbiota, which allows *C. difficile* to proliferate.^1^

*C. difficile* produces up to three exotoxins (toxin A (TcdA), toxin B (TcdB), and the binary toxin CDT), which are the major virulence factors in CDI and are thought to act in synergy to cause inflammation and tissue damage.^6,7^ TcdA and TcdB have similar four-domain structures. The C-terminal domains (called combined repetitive oligopeptide sequences or CROPS) are highly flexible and function as toxin attachment modules required for endocytosis. However, regions outside the C-terminal receptor-binding domains are also involved in cell entry, as CROPS-deleted TcdA and TcdB are still cytotoxic.^8,9^ TcdA is believed to bind multiple cell surface receptors simultaneously, such as sulfated glycosaminoglycans, proteoglycans, and low-density lipoprotein receptors, whereas TcdB is associated with the Wnt receptors Frizzled 1/2/7, the adhesion protein nectin 3, chondroitin sulfate proteoglycan 4 (CSPG4), and possibly other glycan receptors. The delivery domains mediate pore formation and translocation of the toxin across the endosomal membrane, and the autoprotease domains, activated by hexakisphosphate, catalyze cleavage and release of the N-terminal domains in the cytosol. The free N-terminal domains in turn glucosylate host GTPases, leading to loosening of tight junctions and focal adhesion due to disruption of actin filaments, cytokine production, and cell death.^6^ Approximately one-fifth of *C. difficile* strains also produce CDT, whose role in infection is less well understood, although the CDT-producing strains are associated with poor prognosis.^10^ CDT is a binary toxin consisting of CDTb, which is required for cell binding and the formation of pores through which the second component, the actin-specific ADP-ribosyltransferase CDTa, is translocated. In turn, actin cytoskeleton depolymerization is initiated, leading to aberrant microtubule growth and its protrusion of the colonocyte cell membrane, likely facilitating *C. difficile* adherence.^6,11,12^

CDI is still most commonly treated with antibiotics.^13^ The recommended antibiotics for primary and recurrent CDI are vancomycin and fidaxomicin.^14^ However, *C. difficile* spores can survive antimicrobial therapy, and relapse of CDI can occur after germination.^1^ In recent years, administration of microbes has emerged as the second most important treatment option with the goal of restoring microbiota composition and function.^15–17^

**Figure 1.**
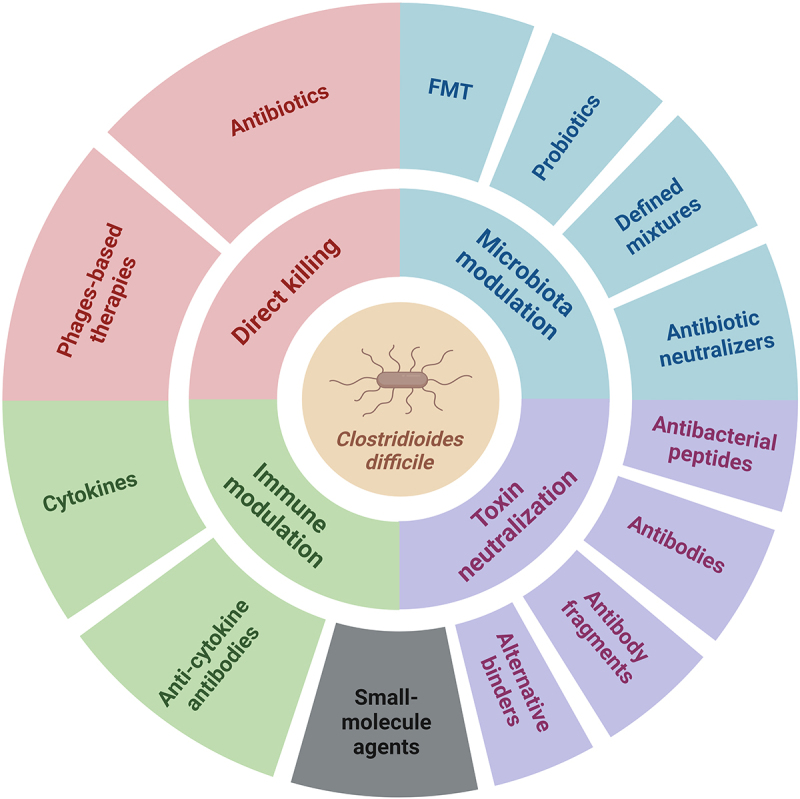
Schematic representation of four different groups of strategies for the treatment of *C. difficile* infections. Antibiotics, fecal microbiota transplantation (FMT), probiotics, and defined mixtures are discussed briefly in this review. The image was created with BioRender.

Despite the considerable success achieved with antibiotics and microbiota-targeted interventions, new approaches are needed, and the particular focus of this manuscript is on their alternatives or complementary strategies that are just entering the field or are already well established. A simplified schematic overview of approaches to treat CDI is shown in Figure 1.

## 2. Administration of microbes for targeted microbiota modification

An imbalanced microbiota is a precondition for *C. difficile* colonization and CDI development and plays an important role in recurrent CDI. Therefore, restoring microbiota diversity and colonization resistance is an important goal for therapeutic and protective approaches.^18^ Fecal microbiota transplantation (FMT) is a widely used treatment option for recurrent CDI in various populations, as described in recent reviews.^15,16^ Despite its very good clinical results, FMT has logistical, safety, and acceptance-related drawbacks, and alternative options are being extensively investigated.^19^ Complex microbiota-based live biotherapeutics, still based on fecal material, can be positioned in the intersection between FMT efficacy and probiotics safety and acceptability. The recent FDA approvals of the fecal microbiota products (Rebyota, November 2022; SER-109, April 2023) to prevent the recurrence of CDI are a major step forward. Rebyota (RBX2660) is a standardized material prepared from stool donations from qualified healthy individuals and is administered rectally as a single dose. It has demonstrated a remarkable treatment success rate (78.9%) in patients with recurrent CDI.^20^ SER-109 contains only a purified sporogenic fraction and reduced CDI recurrence from 40% (placebo) to 12% (SER-109).^21,22^ MET-2 is an encapsulated formulation of 40 lyophilized bacterial species that is administered orally and has been shown to be effective and safe in a phase I clinical trial.^23^

On the other hand, non-FMT microbial interventions are also widely studied, and can be divided into three groups: probiotics, the use of non-toxigenic *C. difficile*, and the development of controlled microbial biotherapeutics. Probiotics have been a much-discussed topic in the treatment and prevention of CDI in the past. Several studies have examined the effects of potentially beneficial bacteria on *C. difficile* colonization, growth, or toxin production.^24–30^ In clinical trials, probiotics have been studied as adjunctive therapy for CDI in combination with standard-of-care antibiotics.^31^ The results were inconclusive as to whether probiotics are effective in preventing CDI. The current guidelines state that there is insufficient evidence to recommend the use of probiotics for the primary prevention of CDI or for the prevention of CDI recurrence.^13,32^ The main problems were the differences in study design (different bacterial strains, dose, duration of treatment), the small number of participants, strain-specific effects, and unknown quality and composition of probiotic products. CDI recurrence was a primary outcome in a small number of randomized controlled trials on probiotics.^33^ In most cases, CDI was a secondary outcome, and thus the trials were not sufficiently powered to detect a statistically significant benefit. Several meta-analyses have been performed and found “moderate quality evidence” that probiotics can prevent CDI.^25^ While probiotics based on single strains gave mixed results, better therapeutic outcomes were reported when probiotics with multiple strains were used.^24–26^ The number of reports of interactions between individual intestinal isolates or commercially available probiotic strains and *C. difficile* is considerable and beyond the scope of this review. Instead, the reader is referred to recent reviews on this topic.^27–30^ In future research, robust, randomized, controlled clinical trials (with defined probiotic strain or combination of strains at appropriate doses) are needed to fill the scientific gaps regarding the potential protective effect of probiotics on CDI. Confirmatory studies with the same strains and a risk-benefit analysis should be performed. Although probiotics are generally considered safe, they may pose some safety risk in vulnerable patient groups such as immunocompromised patients.^34^ Therefore, safety assessment should always be included in the design of clinical trials with probiotics.

The observation that prior colonization with nontoxigenic (NTCD) *C. difficile* strains can protect hamsters (which are more susceptible to CDI than mice) from subsequent colonization with a toxigenic pathogenic *C. difficile* strain was first made around 40 y ago,^35,36^ and considerable progress was made since then. Spores of NTCD strains M3, M23, and T7 were administered to hamsters pretreated with clindamycin and shown to colonize the intestine. After 5 d, hamsters were inoculated with spores of toxigenic *C. difficile* strains, and the non-toxigenic strains prevented disease in 87%−97% of hamsters.^37^ Apart from clindamycin, pretreatment of hamsters with ceftriaxone and ampicillin was tested by the same authors. Again, administration of a single dose of NTCD spores enabled the survival of 100% of hamsters pretreated with ceftriaxone. In contrast, multiple doses of spores were needed for effective colonization and protection of hamsters following pretreatment with ampicillin, to which NTCD and pathogenic strains were both susceptible.^38^ Based on these studies, increasing doses of NTCD strain M3 (NTCD-M3) spores were tested in healthy adult volunteers. NTCD-M3 was well tolerated and was able to colonize the intestinal tract of volunteers pretreated with vancomycin.^39^ This study was upgraded in a phase II placebo-controlled, double-blind, randomized trial on 168 patients with CDI who clinically recovered following treatment with metronidazole or vancomycin.^40^ Administration of NTCD-M3 spores was well tolerated and safe, and colonization occurred in 69% of patients. Recurrence of CDI decreased with NTCD-M3 treatment, and occurred in 11% of NTCD-M3 patients vs. 30% of placebo patients. Recently, the colonization ability of NTCD-M3 was also demonstrated in hamsters pretreated with fidaxomicin, current drug of choice for CDI, supporting the initiation of a phase III clinical trial.^41^ Apart from NTCD, exclusion of pathogenic *C. difficile* strains can also be achieved by genetically engineering probiotic lactic acid bacteria which are considered safe if ingested. Two lactic acid bacteria, *Lactobacillus casei* strain 334 and *Lactobacillus acidophilus* strain 4356, were engineered to display on their surface a fusion protein consisting of a fragment of Surface layer protein A (SlpA) of *C. difficile* and the cell wall anchor of Surface layer protein (Slp) from *L. acidophilus* with the idea of competitively excluding *C. difficile* from the intestinal surfaces by competing for the same adhesion sites. The fusion protein was detected on the lactobacilli surface, and the lactobacilli were safe and able to colonize the intestinal tract of hamsters and piglets. Moreover, the engineered lactobacilli were able to protect hamsters from *C. difficile* induced death.^42^

## 3. Antibodies and antibody alternatives for neutralization of C. difficile toxins

**Figure 2.**
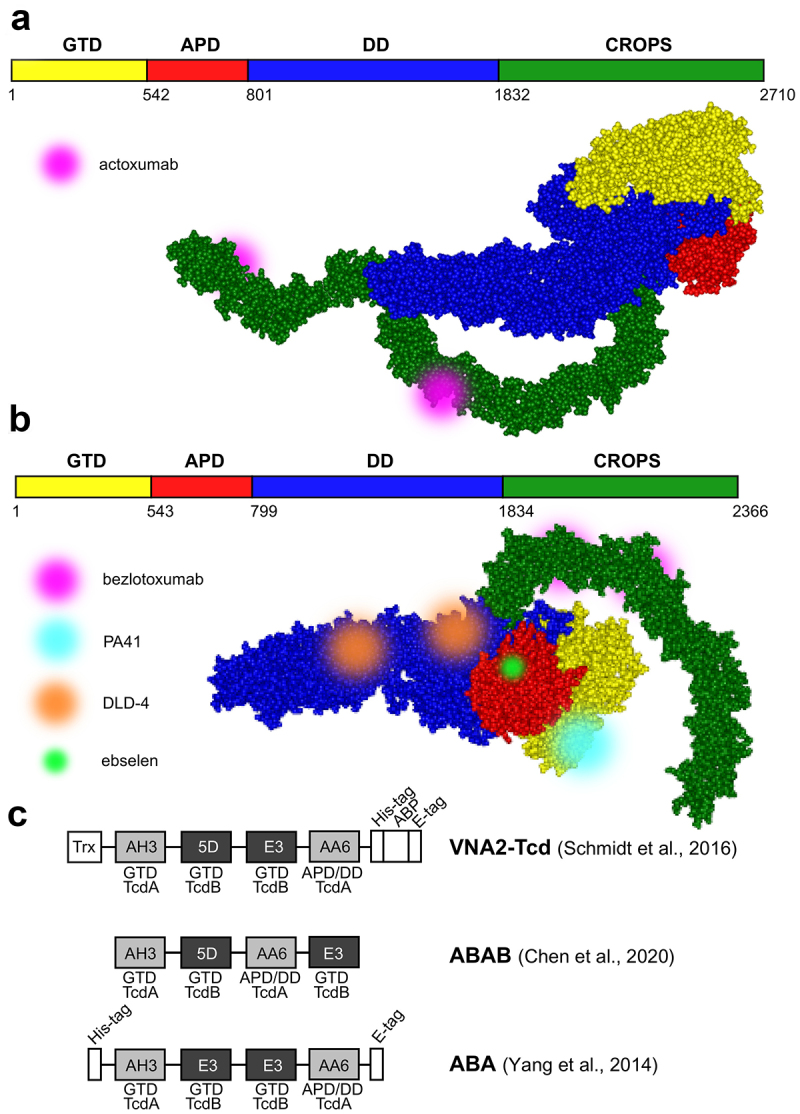
Selected TcdA and TcdB toxin-neutralizing antibodies and antibody alternatives. a. and b. Schematic representation and structural models of TcdA (merged from PDB IDs: 7POG and 2QJ6) and TcdB toxins (PDB ID: 6OQ5) with labeled binding sites of monoclonal antibodies actoxumab, bezlotoxumab, and PA41, the DLD-4 DARPin dimer, and small molecule compound ebselen. Toxin structures were visualized using ViewerLite 4.2 (Accelrys). GTD – glucosyltransferase domain, APD – autoprotease domain, DD – delivery domain, CROPS – combined repetitive oligopeptide sequences (receptor-binding domain). c. Schematic structures of various tandem V_H_H constructs (nanobodies). Individual nanobodies bind to distinct epitopes on the TcdA (light gray) or TcdB (dark gray) domains. Trx – thioredoxin, ABP – albumin-binding peptide.

Antibodies are a critical tool of our immune system in the fight against microbial infections and an important part of our therapeutic arsenal in general, with monoclonal antibodies making up the majority of approved biopharmaceuticals. In addition to monoclonal antibodies already in clinical use for the treatment of CDI, polyclonal antibodies, antibody fragments, and alternative binders have also been tested. Given the importance of *C. difficile* toxins in CDI pathogenesis, most antibody therapies target toxins and are designed to effectively limit *C. difficile* colonization of the gut following antibiotic treatment. Indeed, numerous preclinical studies have confirmed that neutralization of single or multiple *C. difficile* toxins prevented cytotoxicity in cell assays and protected animals from toxin/spore challenge (e.g.,^43–46^), as well as facilitated normalization of the gut microbiota in CDI. However, it seems that an interplay of different factors, such as the targeted epitope, binding affinity, and stoichiometry (as well as avidity and molecular topology for constructs with more than one paratope) determine the neutralization potency.^47^ Table 1 provides an overview of antibodies and antibody alternatives, and selected examples are shown in Figure 2.

**Table 1.** Antibodies and antibody alternatives for neutralization of *C. difficile* toxins.

Agent	Target	Stage of development	Delivery route	Reference
Bezlotoxumab	TcdB	Approved	intravenous (infusion)	^48–50^
Actoxumab	TcdA	Phase III, discontinued	intravenous (infusion)	^50,51^
PA41 (monoclonal antibody)	TcdB	Preclinical (cell model)	/	^52^
Three-monoclonal-antibody cocktail	TcdA and TcdB	Preclinical (mice, hamsters)	intraperitoneal (injection)	^53,54^
Horse antiserum	*C. difficile* spores and TcdA/TcdB toxoids	Preclinical (mice)	intravenous (injection)	^55^
OraCAb (polyclonal anti-TcdA/TcdB ovine antibody formulation containing antacids and protease inhibitors)	TcdA and TcdB	Preclinical (hamsters)	oral	^56^
WPC-40 (hyperimmune whey protein concentrate from cow)	*C. difficile* cells and toxins	Uncontrolled human clinical trial	oral	^57,58^
TcdB-specific bovine colostrum	TcdB	Preclinical (mice)	oral	^59^
Hyperimmune bovine whey protein concentrate	TcdA and TcdB	Preclinical (mice, hamsters)	oral	^60,61^
Nanobodies and nanobody fusions	TcdA and TcdB	Preclinical (mice, hamsters, piglets)	oral (via engineered *Lactobacillus paracasei*); oral (via engineered *Saccharomyces boulardii*; intravenous (via adenoviral vector); intraperitoneal or intramuscular (injection)	^62–67^
DARPins and DARPin fusions	TcdA and TcdB	Preclinical (mice)	oral (gastrointestinal stability only)	^68–70^

### 3.1. Monoclonal antibodies

To date, bezlotoxumab is the only add-on anti-toxin therapeutic approved for the prevention of CDI recurrence in high-risk adults undergoing antibiotic therapy. Bezlotoxumab is a fully human IgG1 monoclonal antibody against the TcdB CROPS domain, where it binds two adjacent epitopes (Figure 2b) and blocks the interaction of TcdB with CSPG4.^48^ Interestingly, the TcdB-neutralizing activity is mediated via an allosteric mechanism, as bezlotoxumab induces a conformational change in TcdB, thereby masking the CSPG4 binding site.^71^ Bezlotoxumab was developed by hybridoma technology using mice transgenic for human immunoglobulin genes that were immunized with TcdA and TcdB toxoids and recombinant C-terminal TcdB fragment.^72^ It is administered as a single intravenous infusion during the course of antibacterial therapy. Considering that CSPG4 is expressed on cells of the subepithelial layer rather than on colonocytes, bezlotoxumab is thought to be transported to the luminal side of the intestinal epithelium by paracellular transport after toxin-induced breach of the epithelial barrier.^73^ Therefore, bezlotoxumab has been suggested to be especially useful for the treatment of severe CDI episodes.^49^

The efficacy of bezlotoxumab was evaluated in two phase III placebo-controlled, double-blind, randomized trials (MODIFY-I and MODIFY-II) involving a total of 2655 adult patients receiving standard-of-care oral antibiotics for primary or recurrent CDI.^50^ Patients were randomized in a 1:1:1:1 ratio to receive either a single infusion of bezlotoxumab (10 mg/kg), actoxumab (10 mg/kg), bezlotoxumab plus actoxumab (10 mg/kg each), or placebo. Actoxumab is another monoclonal antibody that targets TcdA and was used only in MODIFY-I. In *in vitro* cell survival assays, actoxumab alone fully neutralized TcdA from culture supernatants of various clinical isolates.^74^ However, the actoxumab group in MODIFY-I was discontinued after an interim analysis due to lack of efficacy and was not initiated in MODIFY-II. In both trials, the percentage of patients who suffered recurrent CDI during the three-month follow-up period was lower in the bezlotoxumab-treated group than in placebo (MODIFY-I: 17% vs. 28%; MODIFY-II: 16% vs. 26%; both *p* < .001). Combined actoxumab/bezlotoxumab treatment showed similar efficacy (MODIFY-I: 16% vs. 28% (placebo); MODIFY-II: 15% vs. 26% (placebo); both *p* < .001), while actoxumab treatment alone turned out to be ineffective (26% vs. 28% (placebo); *p* = .64). A meta-analysis^75^ of post-marketing data from 11 observational and 2 controlled clinical trials on a total of 2337 patients with CDI (of which 1472 were treated with bezlotoxumab) confirmed the level of bezlotoxumab’s efficacy similar to the one determined in MODIFY trials, as well as suggested its cost-effectiveness when added to standard-of-care therapy. Interestingly, bezlotoxumab may exert an additional mechanism of action as inferred from a recent animal study.^76^ In spore-challenged mice, bezlotoxumab intraperitoneal injection prevented toxin-mediated systemic disease arising from the dissemination of luminal contents to other organs leading to cytokine induction. Liver and kidney function failure, but also thymic atrophy and a reduction in CD4+/CD8+ thymocyte numbers were effectively counteracted with bezlotoxumab. The authors speculated that the antibody’s efficacy in preventing recurrent CDI may be in part due to its ability to preserve the critical naive effector T cell pool required to fend off the infection.

Other potent and broadly-neutralizing anti-toxin mAbs have been reported (reviewed in^6^). Of these, PA41 deserves special attention as it exhibits a unique mechanism of action.^52^ Namely, PA41 binds to the N-terminal glucosyltransferase domain of TcdB (Figure 2b) and blocks its translocation across the endosomal membrane. Due to the poorly defined role of the binary toxin in the pathophysiology of CDI, there were few attempts to design CDT-neutralizing antibodies. An interesting example was recently reported by Goldsmith et al.^77^ who immunized mice with the monomeric CTDb to develop antibodies interfering with CTDb oligomerization, preventing assembly of the di-heptameric pores required for the delivery of catalytic CTDa toxin.

### 3.2. Limitations of using monoclonal antibodies for therapy and possible solutions

Despite its clinical use, there are some limitations of monoclonal antibodies that should be considered. Because many hypervirulent *C. difficile* strains harbor mutations in the TcdB epitopes of bezlotoxumab, the monoclonal antibody likely exhibits a limited range of neutralizing activity. In addition, bezlotoxumab is not expected to interfere with the binding of TcdB to Nectin 3 or Frizzled 1/2/7 receptors. Considering the risk of escaping mutations in epitopes of monoclonal antibodies that would render mAbs ineffective, a competitive approach based on toxin receptor fragments could provide ‘sturdy’ anti-toxin therapeutics with broad-spectrum activity. For example, a CSPG4 receptor decoy was developed by fusing one of the extracellular CSPG4 repeat domains to the Fc region of IgG.^71^ The artificial decoy receptor completely blocked two diverged TcdB variants in the *in vitro* cell assay and significantly outperformed bezlotoxumab in neutralizing TcdB2 (the toxin isoform expressed by the hypervirulent ribotypes BI/NAP1/027).

The lack of efficacy of actoxumab can be explained in part by the fact that this antibody prevents the binding of TcdA to cell surface receptors by directly blocking only two of the putative seven carbohydrate-binding sites within the CROPS domain.^51^ Moreover, actoxumab cannot bind both epitopes at the same time as they are located on opposite sides of the CROPS domain of TcdA (Figure 2a) and therefore affects the conformation of the toxin to a lesser extent than bezlotoxumab. Indeed, actoxumab induces TcdA aggregation, which is due to one antibody molecule binding to two different toxin molecules, whereas bezlotoxumab does not form higher-order immunocomplexes with TcdB. Other regions (inside and/or outside the CROPS domain) might be involved in the entry of TcdA into the cell, and anti-TcdA antibodies targeting other epitopes (or perhaps a combination of two or more such antibodies) might confer better protection against TcdA. Conversely, the toxins are not fully conserved among different strains. Both, actoxumab and bezlotoxumab were shown to display a broad range of neutralization potencies against exotoxins of different ribotypes on account of targeted epitope variability.^74^ In line with this, Cole et al.^78^ have shown that a mixture of monoclonal antibodies (mAbs) targeting different toxin domains had a higher neutralizing potency than single mAbs or a combination of mAbs specific for one domain. Through careful selection, Davies et al.^53^ and Qiu et al.^54^ each developed a cocktail of three monoclonal antibodies against TcdA and TcdB that neutralized the toxins highly efficiently in cell-based *in vitro* assays and provided hamsters with a high level of protection during *C. difficile* challenge (in one of the studies, the efficacy was compared head-to-head with that of actoxumab/bezlotoxumab combination and was found to be superior).

Apart from the limitations discussed above, the prospects of monoclonal and polyclonal antibodies *parenteral* use may face the same limitations as all the *C. difficile* vaccines evaluated in clinical trials thus far. While the toxoids were effective in raising systemic antibody responses and reduced symptomatic disease, they had little effect in preventing the persistence of *C. difficile* in the host,^79^ likely due to poor gut mucosal immunity. Delivering toxin-neutralizing agents to the gut (the site of infection) may be required for achieving optimal therapeutic effects; however, designing appropriate drug formulations is challenging.

### 3.3. Polyclonal antibodies

The idea of harnessing polyclonal or oligoclonal anti-toxin antibody repertoires to achieve strong toxin neutralization has been explored by several groups. For example, Yan et al.^55^ produced an antiserum by immunizing horses with inactivated *C. difficile* spores and TcdA/TcdB toxoids and showed that this antiserum protected mice from CDI in a dose-dependent manner when administered intravenously. Notably, mice receiving the prophylactic antiserum lost less weight, showed no signs of infection, and their weight normalized more rapidly than control animals treated with the preimmune serum.

To avoid parenteral administration and instead deliver neutralizing antibodies directly to the intestinal lumen where toxins are produced, special oral formulations or hyperimmune colostrum and whey protein isolate were considered. OraCAb is a highly concentrated ovine polyclonal anti-TcdA/TcdB antibody formulation containing antacids and protease inhibitors from dried hen egg white to protect immunoglobulins from denaturation at low pH and degradation by pepsin, trypsin, and chymotrypsin during transit from the mouth to the large intestine.^56^ Neutralizing IgGs were obtained by separately immunizing sheep with nontoxic recombinant fragments of TcdA (TxA4) and TcdB (TxB4), and mixed at an optimized ratio of 1:3 (anti-TxB4 vs. anti-TxA4). OraCAb afforded significant protection to hamsters against CDI. Animals received the OraCAb formulation twice daily for 5 d after oral spore challenge; survival rates 5 d after challenge were 20% in the untreated group, 40% in the anti-TxB4-treated group, and 100% in both the optimized (1:3) and the 1:1 anti-TxB4:anti-TxA4 OraCAb formulation-treated groups. At the end of the study (day 15), survival rates were similar (20–30%) in the untreated, anti-TxB4-only, and 1:1 anti-TxB4:anti-TxA4 OraCAb-treated groups, whereas 60% of animals in the group treated with the optimized OraCAb formulation survived infection. Furthermore, in an *in vitro* gut model of CDI seeded with human fecal inoculum, OraCAb completely neutralized the toxins and had no effect on the composition of the colon microbiota. Protein-rich colostrum and whey possess buffering capacity and likely provide alternative substrates for proteases, thereby protecting immunoglobulins from inactivation in the gastrointestinal tract.

Van Dissel et al.^57^ produced a hyperimmune whey protein concentrate (called WPC-40) from milk of cows immunized with formaldehyde-inactivated whole *C. difficile* cells and toxoid from bacterial culture filtrate. Secretory IgA was the dominant specific immunoglobulin isoform in the whey with a 100-fold higher titer compared to IgG. WPC-40 was administered to hamsters challenged with *C. difficile* via a feeding tube three times daily for 3 d, and the animals were monitored for a total of 14 d. The survival rate of hamsters receiving WPC-40 or its 10-fold concentrate was 90% and 80%, respectively, whereas none of the animals treated with preimmune whey survived. Notably, 20% of hamsters treated with a concentrated control whey preparation survived, suggesting that nonspecific bactericidal proteins from milk (e.g., lysozyme and lactoferrin) confer some degree of protection. Encouraged by these results, the team conducted two small uncontrolled clinical trials to preliminarily evaluate the efficacy of WPC-40 in eradicating CDI. In the first trial,^57^ 16 patients with *C. difficile* diarrhea (9 with recurrent disease) received immune WPC-40 three times daily for 2 weeks after standard antibiotic treatment. With one exception, no *C. difficile* toxins were detected in any of the patients’ stools at the end of treatment, and none of the patients reported another episode of *C. difficile* diarrhea during the median follow-up period of 333 d (range: 35 d to 1 y). The second study^58^ enrolled 101 patients with *C. difficile*-associated diarrhea who received immune WPC-40 using the same dosage regimen, 8 of whom did not complete treatment. The relapse rate of *C. difficile* diarrhea during the 60-d follow-up period was 10%, which was significantly lower compared with the values of local contemporary controls and published data for the same prevalent *C. difficile* ribotype 027 (20–25% and 20–47%, respectively).

Oral immunotherapeutics based on bovine immune colostrum or protein-enriched whey have also been evaluated for the treatment of CDI and prevention of its recurrence. Hutton et al.^59^ have shown that TcdB-specific bovine colostrum alone or in combination with a preparation targeting spores or vegetative cells can be used for both prevention and treatment of CDI in a mouse model of infection. Whereas most mice (89%) that did not receive colostrum died 8–14 d after vancomycin was discontinued, the mortality rate was significantly lower (22%) in mice treated with the mixture of immune colostra. Animals in this group still had mild diarrhea and shed *C. difficile* spores, indicating that colostrum antibodies did not prevent bacterial colonization, but effectively neutralized TcdB. In contrast, Heidebrecht et al.^60^ reported that short-term (75 h) treatment of hamsters with hyperimmune bovine whey protein concentrate, produced by immunizing cows with TcdA/TcdB toxoid and inactivated *C. difficile* cells/spores, not only neutralized the toxins, thus suppressing the initial CDI, but also effectively limited *C. difficile* growth, thereby preventing disease recurrence. Furthermore, this formulation enabled instant regeneration of the intestinal microbiome, in sharp contrast to treatment with vancomycin, which significantly perturbed the microbiota.^61^ Despite the attempts to address the issue of oral delivery, batch variability and formulation of appropriate dosage form remain the limitations of this system.

### 3.4. Antibody fragments

Because toxin inactivation seems to rely on direct neutralization by antibodies and does not require Fc-mediated effector functions, antibody fragments, such as nanobodies (single-domain antibodies engineered from camelid heavy-chain antibodies or V_H_Hs), are an attractive alternative to full-length mAbs as antitoxin therapeutics. Their simplified but stable structure is compatible with microbial production, and there are well-established platforms for nanobody development.

Recently, Kordus et al.^80^ have demonstrated that no exotoxin domain is immunodominant by isolating a panel of nanobodies binding to different structural/functional segments of TcdA and TcdB from a phage-displayed nanobody library constructed from alpacas immunized with nontoxic variants of both toxins. The majority of the neutralizing nanobodies bound the autoprotease/delivery and the receptor-binding domains, but some TcdB neutralizers targeted the glucosyltransferase domain. In an earlier report, Hussack et al., using a similar approach but immunizing llamas with TcdA and TcdB CROPS domains,^62^ identified several selective V_H_H binders. Most of the anti-TcdA nanobodies recognized conformational epitopes located outside the carbohydrate-binding pockets of the toxin and, in combination, exhibited synergistic neutralizing activity. When two such V_H_Hs (termed A20 and A26) were fused via a flexible peptide linker,^81^ the biparatope construct exhibited a drastically increased TcdA neutralizing activity. Interestingly, the A26-A20 construct (in which the order of nanobodies was reversed) was a much weaker toxin neutralizer *in vitro* (with a 29,000-fold difference in IC_99_ values). Whereas the A20-A26 construct bound a single toxin molecule with both V_H_H domains simultaneously (thus displaying avidity), the reverse orientation of nanobodies precluded the 1:1 type interaction and led to formation of tri- and tetrameric complexes. This indicates the importance of topological arrangement of antibody fragments when designing toxin neutralizers aimed at concurrent binding of more paratopes to a single target molecule. It is not clear whether the A20-A26 construct exerts superior neutralization activity simply via occupying the carbohydrate-binding sites on TcdA, or perhaps inhibits the pH-induced toxin conformational changes. In contrast, none of the originally reported anti-TcdB nanobodies were able to neutralize the toxin *in vitro*.^62^ Later, the same group reported the identification of a large set of TcdB-binding nanobodies that still failed to neutralize the toxin despite much higher affinity.^63^ However, after grafting the nanobodies onto the IgG Fc region (i.e., V_H_H-Fc fusions), these constructs showed neutralizing activity *in vitro* comparable to that of bezlotoxumab. This observation can be explained by the assumption that, once again, divalent binding is required for the induction of nonproductive toxin conformation. Alternatively, nanobodies, if presented in a divalent setting, would be expected to induce agglutination of toxin, whereas a monomeric V_H_H would be unable to clump TcdB.

Of note, Andersen et al.^64^ developed several unique anti-TcdB nanobodies, two of which, while not neutralizing the toxin when expressed in soluble monomeric form, effectively adsorbed TcdB when displayed on the surface of lactobacilli and thus afforded protection in an *in vitro* cell assay. The use of lactic acid bacteria as a delivery system for antitoxins is a promising strategy because these microorganisms are known to survive passage through the gastrointestinal tract, allowing production of neutralizing agents at the site of infection. A combination of two orally administered *Lactobacillus paracasei* strains expressing two surface-anchored neutralizing anti-TcdB nanobodies that recognize nonoverlapping epitopes protected hamsters after a challenge with *C. difficile* TcdA^–^ TcdB^+^ strain spores, whereas isolated recombinant nanobodies offered no protection.^64^ Five days after spore challenge, all animals in the control groups (receiving no treatment or non-expressing lactobacilli) died, whereas 50% of animals receiving lactobacilli expressing toxin-neutralizing nanobodies survived, and showed no behavioral signs of infection and only limited inflammation of the colonic mucosa despite colonization with *C. difficile*.

Similarly, Chen et al.^65^ used the probiotic yeast *Saccharomyces boulardii* as a biological delivery system for antitoxin nanobody fusions compatible with oral administration. Broadly-neutralizing tetra-specific four-V_H_H-fusion (termed ABAB, consisting of two nanobodies against TcdA and two against TcdB; similar constructs dubbed VNA2-TcD^66^ and ABA^82^ (Figure 2c) were previously shown to reverse fulminant disease symptoms in mice with CDI when administered parenterally) were produced *in situ* by orally administered constitutively secreting engineered yeast strain. ABAB demonstrated both prophylactic and therapeutic efficacy against CDI and primary/recurrent CDI, respectively, in mice and hamsters. Importantly, as yeast is not affected by antibacterial agents (in contrast to lactic acid bacteria), the treatments can be concurrent with antibiotics commonly prescribed for CDI. The anti-TcdA V_H_Hs used in the construction of ABA^82^ display unusual neutralizing mechanisms as demonstrated later^83^: AH3 targets the glucosyltransferase domain to enhance its stability and interfere with its unfolding at acidic endosomal environment, whereas AA6 binds to the delivery domain and is presumed to inhibit its conformational changes required for pore formation.

### 3.5. Adenoviral delivery of antibodies and antibody fragments

Gene therapy approaches have been explored to achieve long-term efficacy of antitoxin therapy that would provide protection against recurrent disease. Schmidt et al.^66^ and Yang et al.^67^ used replication-incompetent adenoviral vectors to deliver tetravalent bispecific nanobody constructs against TcdA and TcdB (Figure 2c). High levels of serum antitoxins were detected in gnotobiotic piglet and mouse models, protecting the animals from oral *C. difficile* spore and systemic toxin challenge, respectively. Treated pigs suffered only mild-to-moderate diarrhea and had less severe histopathologic lesions in the large intestine. In contrast, 50% of the control animals had severe diarrhea and systemic signs of CDI, including pleural effusions and ascites. In treated animals, there was a significant negative correlation between the severity of symptoms and serum levels of viral vector-induced antitoxin.^66^ Mice challenged with *C. difficile* spores and given a single dose of the adenoviral vector during antibiotic treatment were fully protected from both primary and recurrent CDI, whereas the survival rate of animals from the control group was 30% after initial challenge and 70% after re-challenge.^67^ Recently, Guilleman et al.^84^ reported the use of two adeno-associated viral vectors to deliver actoxumab and bezlotoxumab to mice and hamsters. Both antibodies were present in serum in high titers and could be detected on mucosal surfaces after a single intramuscular administration of the viral vectors. Vector-delivered actoxumab protected mice from systemic TcdA challenge, whereas vector-mediated expression of bezlotoxumab failed to protect against TcdB challenge. Rather, mice expressing bezlotoxumab were more susceptible to TcdB. The authors speculated that the higher mortality observed in the treated group might be caused by bezlotoxumab-enhanced toxin uptake in the mouse host cells because of the high affinity of the human IgG Fc region for mouse Fc gamma receptors.

### 3.6. Alternative binders

Artificial binding proteins engineered from non-immunoglobulin scaffolds using *in vitro*-molecular evolution approaches^85^ are considered a viable alternative to antibodies and antibody fragments. Designed ankyrin-repeat proteins (DARPins), for example, have a favorable immunogenic profile, exhibit high thermostability and solubility, bind various targets with specificity and affinity comparable to antibodies, and can be expressed at high levels in *Escherichia coli*.^86^ DARPins are single-domain proteins with a modular structure of two to four internal ankyrin repeats (each consisting of two alpha helices separated by loops) flanked by *N*- and C-terminal capping repeats. The surface-exposed residues of the internal repeats can be randomized to prepare a combinatorial DARPin phage display library that is subjected to affinity selection against a selected target. Using this approach, Simeon et al.^68^ identified a panel of TcdB-binding DARPin variants. In the next step, a library of anti-TcdB DARPin dimers was constructed and functionally screened for toxin-neutralizing activity using a cell assay. The most effective construct, termed DLD-4 (1.4E/U3 dimer), contained domains targeting the central (1.4E) and C-terminal (U3) regions of the toxin’s delivery domain (Figure 2b) and blocked the interaction of TcdB with CSPG4 and Frizzled 2 receptor. DLD-4 exhibited neutralizing effect against TcdB from ribotypes 0120 and 087 *in vitro* that surpassed that of bezlotoxumab by one to two orders of magnitude (33- and 330-fold, respectively) and significantly protected mice from toxin challenge when injected intraperitoneally at the same times as TcdB. The same group also reported the development of DARPins capable of neutralizing TcdB from the hypervirulent 027 strains.^69^ The most potent monomeric DARPin, D16, interfered with the binding of TcdB to CSPG4 and neutralized the TcdB isoform of ribotype 027 more efficiently than bezlotoxumab *in vitro*. When fused with U3 DARPin targeting Frizzled 1/2/7-binding toxin epitopes, the dimeric construct neutralized TcdB of the 017 ribotype strain *in vitro* with high efficiency, but showed identical activity against the 027 ribotype toxin compared with the D16 monomer. This was later explained by the lack of binding of 027 ribotype TcdB to the Frizzled 1/2/7 receptors, highlighting the importance of targeting multiple epitopes in the development of broad-acting antitoxin therapeutics. In an attempt to improve the resistance of DARPins to gut-resident proteases that would allow oral administration, 1.4E was later re-engineered by mutating the trypsin- and chymotrypsin-sensitive residues.^70^ The resulting DARPins retained similar anti-TcdB neutralizing activity *in vitro* as their parent, while exhibiting increased chemostability (judging from their recovery from mouse fecal samples upon oral administration).

Apart from DARPins, affimers evolved from the protease inhibitor Stefin A using phage display are another group of non-immunoglobulin binders that have been selected against distinct epitopes of TcdB. However, their reported use was not for exotoxin neutralization, but rather for diagnostic applications, using a split-luciferase assay in combination with a nanobody.^87^ The affimer was fused via a flexible peptide linker to the large subunit of luciferase (18 kDa, termed LgBiT), and the nanobody was tethered to the small subunit (the 11-residue SmBiT). Upon simultaneous binding of both constructs to TcdB, the SmBiT subunit complemented LgBiT to form catalytically active luciferase, thus producing a luminescent signal. The sensor design required careful optimization (i.e., the constructs were sensitive to topological organization, such as fusion of binding modules to *N*- or C-termini of luciferase subunits and specific combinations of luciferase subunits and binding modules). Using this assay, the authors could detect TcdB in the stool sample matrix down to the concentration of 2 pM.

## 4. Antibacterial peptides against C. difficile toxins

Antimicrobial peptides are natural peptides found in prokaryotes and eukaryotes, including insects, humans, and microorganisms. They are involved in innate immunity and have a broad spectrum of antimicrobial activity. The secondary structures and electrostatic properties of peptides play an important role in their activity. Most antimicrobial peptides are positively charged and act by forming a pore in the bacterial cell envelope, which depolarizes the membrane and leads to cell death. It has been found that α-helical and *β*-sheet peptides rich in cysteine residues have potent antibacterial activity.^88^ However, in the case of *C. difficile*, peptides mostly exert nonspecific activity by directly targeting toxins and blocking their deleterious effects on epithelial cells (Table 2). The reports on antimicrobial peptides are proof-of-principle studies, where the peptides were mostly evaluated *in vitro* on cell cultures and/or on isolated ileal loops. Additional studies are therefore needed to substantiate their further development.

**Table 2.** Antibacterial peptides against *C. difficile* toxins.

Agent	Target	Stage of development	Delivery route	Reference
α-defensin-1	TcdA, TcdB and CDTb	Preclinical (mice)	injection in isolated ileal loops	^89^
α-defensin-5	TcdA, TcdB and CDT	Preclinical (*in vitro* assay)	/	^46^
Periplanetasin-2	TcdA	Preclinical (mice)	injection in isolated ileal loops	^90^
Periplanetasin-4	TcdA	Preclinical (mice)	injection in isolated ileal loops	^91^
Cathelicidin LL-37	TcdA	Preclinical (mice)	injection in isolated ileal loops; intracolonic administration	^92^

Defensins, conserved small cationic peptides stabilized by three intramolecular disulfide bonds, are well known for their antimicrobial activity and neutralization of bacterial exotoxins, including those of *C. difficile*.^46,89,93^ Using *in vitro* cell assays, Fischer et al.^89^ demonstrated that human α-defensin-1 inhibits TcdA, TcdB, and CDTb by direct neutralization. Mechanistic analyses revealed that α-defensin-1 not only caused local unfolding of the thermodynamically unstable TcdA/TcdB regions,^94,95^ but also induced aggregation of TcdA and inhibited the formation of CDTb channels to prevent CDTa entry into cells. Furthermore, α-defensin-1 specifically protected Vero and Caco-2 cells and human intestinal organoids from all three toxins and was active *in vivo* in preventing TcdA-induced damage to intestinal loops in mice. Similarly, Korbmacher et al.^46^ confirmed the specific antitoxin activity of human α-defensin-5 against TcdA, TcdB, and CDT *in vitro*.

Abnormally increased apoptosis in mucosal epithelial cells is the first step of the inflammatory response to *C. difficile* in the gut affected by pseudomembranous colitis. Peptides that block toxin A-induced apoptosis of mucosal epithelial cells have been tested for their potential to suppress excessive inflammatory responses. Two short antimicrobial peptides identified by transcriptome analysis of the American cockroach (*Periplaneta americana*), termed periplanetasin-2^90^ and periplanetasin-4,^91^ have been shown to inhibit TcdA-induced apoptosis in cultured human colon tumor cells and ameliorate TcdA-induced enteritis in a mouse intestinal loop model. The molecular mechanism of action of periplanetasins was not fully elucidated, but the two peptides significantly decreased reactive oxygen production (a hallmark of TcdA exposure), leading to suppression of p38MAPK activation and thus decreased expression of cyclin-dependent kinase inhibitor 1 (p21^cip1/waf1^), an essential mediator of apoptosis in colonic epithelial cells. In mouse ileal loops, intravenous administration of the peptides concomitantly with TcdA resulted in less mucosal damage and decreased production of the proinflammatory cytokine IL-6, suggesting that periplanetasins may also directly suppress immune cell-mediated inflammatory responses.

The antimicrobial peptide of particular interest, human cathelicidin LL-37, produced by epithelial cells, exerts a bactericidal activity by breaking membrane integrity, and it has been shown to inhibit *C. difficile* proliferation *in vitro*.^96^ It also possesses anti-inflammatory properties and, after intracolonic administration, was able to reduce apoptosis and alleviate TcdA-associated intestinal inflammation (and thus colitis) in a murine *C. difficile* challenge model.^92^ Treatment with exogenous cathelicidin reduced tissue levels of myeloperoxidase and tumor necrosis factor in the colon and was equally effective in reducing these inflammatory mediators in toxin A-exposed ileal loops.^92^

## 5. Neutralization of antibiotics in the colon lumen reduces the risk of C. difficile infections

Treatment of infections with antibiotics results in considerable amount of the active drug reaching the colon lumen and affecting the composition of microbiota, promoting the spread of antibiotic resistance, and increasing the risk of CDI. A high concentration of antibiotics in the colon may be the result of oral administration or bile secretion in the case of parenteral administration. Local inactivation of antibiotics in the colon lumen (Table 3) therefore represents a rationale for the treatment of CDI.

**Table 3.** Agents for the neutralization of antibiotics in the colon lumen.

Agent	Target	Stage of development	Reference
Enteric-coated formulated activated-charcoal based DAV132	Antibiotics in the colon (e.g. fluoroquinolones)	Phase III	^97–99^
Ribaxamase (β-lactamase; formulated as oral capsules containing enteric-coated pellets)	β-lactam antibiotics	Phase IIb	^100,101^
β-lactamase-secreting *Lactococcus lactis*	β-lactam antibiotics	Preclinical (mice)	^102^

DAV132 is an enteric-coated formulation of an activated-charcoal-based product. It is manufactured by formulating activated charcoal with high surface area (>1,500 m^2^/g) and adsorption capacity (iodine number >1,500 mg/g) into pellets 0.5–1 mm in diameter. These are then coated with a layer of pH-sensitive enteric polymer to obtain enteric-coated DAV132. To provide proof of concept, DAV132 was tested in a randomized, controlled crossover study in 18 healthy volunteers who were orally administered the test drugs 500 mg amoxicillin and 25 mg sulfasalazine.^97^ Amoxicillin is known for its rapid absorption in the proximal small intestine, whereas sulfasalazine is a prodrug that is activated by the commensal microbiota, resulting in the release of sulfapyridine, which is then rapidly absorbed in the colon. DAV132 was compared with uncoated formulated activated charcoal and with water and administered before, after, or simultaneously with the test drugs. The plasma concentration of amoxicillin was reduced by more than 70% when taken with control uncoated formulated activated charcoal, but showed no difference when taken with water or DAV132. In contrast, the reduction in plasma concentration of sulfapyridine was similar (more than 90% compared with water) with both DAV132 and control (uncoated formulated activated charcoal).^97^ This confirms that DAV132 can selectively adsorb drugs in the proximal colon and does not affect drug absorption in the proximal small intestine. This was further supported by a phase II clinical trial that evaluated the safety and efficacy of DAV132 in 243 hospitalized fluoroquinolone-treated patients at risk for CDI.^98^ In the treated group, DAV132 (7.5 g) was administered orally three times daily, whereas no DAV132 was administered in the control group. Treatment with DAV132 was associated with a > 98% reduction in fecal fluoroquinolone levels, less impairment of microbiota diversity, and higher *ex vivo* resistance to *C. difficile* colonization.^98^ DAV132 is being assessed in an ongoing phase III clinical trial focused on the prevention of CDI in patients with newly diagnosed acute myeloid leukemia or high-risk myelodysplastic syndrome treated with intensive chemotherapy.^99^

A similar therapeutic goal can be achieved by administering antibiotic-degrading enzymes. Ribaxamase is an orally administered β-lactamase intended for use with intravenous β-lactam antibiotics (penicillins and cephalosporins). Ribaxamase is produced in *Escherichia coli*, purified, and formulated as 75 mg oral capsules containing enteric-coated pellets. In a double-blind phase IIb clinical trial, 412 patients were randomized in a 1:1 ratio to receive either ceftriaxone and ribaxamase (150 mg, four times daily) or placebo. During the study and within 4 weeks of antibiotic treatment, two patients in the ribaxamase group (1.0%) and seven patients in the placebo group (3.4%) were diagnosed with CDI, resulting in a statistically significant risk reduction (*p* = .045).^100^ When fecal samples obtained in the study were analyzed, Kokai-Kun et al. also observed a significant reduction in changes in antimicrobial resistance genes.^101^

An alternative strategy of β-lactamase delivery was recently presented by Cubillos-Ruiz et al.^102^ They engineered a strain of *Lactococcus lactis* to secrete heterodimeric β-lactamase that assembles extracellularly. Such an experimental setup, consisting of two unrelated genes, does not contribute to horizontal gene transfer and the spread of antibiotic resistance genes. The engineered *L. lactis* was administered orally to mice treated parenterally with ampicillin. The treatment minimized gut dysbiosis without affecting serum ampicillin concentrations and prevented the loss of colonization resistance to *C. difficile*.^102^

## 6. Phage-based therapies against C. difficile infections

The success of personalized phage cocktails in recent clinical case studies^103^ has sparked renewed interest in phage therapy, which has been proposed as one of the solutions to the antimicrobial resistance crisis.^104^ The main advantage of bacteriophages (phages for short) over existing antibiotic treatment for CDI is their narrow spectrum of activity, which would circumvent the problem of gut dysbiosis and CDI recurrence after treatment. Since phages self-replicate at the site of infection, lower and fewer doses are required. Compared to conventional drugs, phage preparations can be produced faster and at a lower cost. Importantly, *C. difficile* phages are stable over a wide pH range,^105^ making them suitable for oral administration.

However, the lack of naturally occurring, strictly lytic *C. difficile* phages poses a major challenge in the development of phage therapy for CDI. All *C. difficile* phages characterized to date are temperate (or lysogenic), which means that they can replicate via a lytic or lysogenic life cycle. During the lytic cycle, the production of virions begins immediately after the phage enters the cell, leading to cell lysis. In contrast, during the lysogenic cycle, the phage genome is integrated into the bacterial chromosome as a prophage and the genes required for the lytic cycle are suppressed. The prophage is replicated together with the bacterial genome and transferred to the daughter cells. Bacteria that carry prophages in their genome are called lysogens. In the presence of certain stimuli (e.g., UV radiation or chemicals), prophages can leave the bacterial chromosome and enter the lytic cycle. Lysogenization often renders bacteria resistant to infection with the same or related phages via superinfection exclusion. Temperate phages contain the gene for the integration of the prophage into the bacterial chromosome (integrase) and its maintenance (repressors of the lytic cycle), which gives them the ability to become lysogenic. The integrase gene has been identified in all *C. difficile* phages whose genomes have been sequenced.^106^ This is attributed to the fact that *C. difficile* often occurs in the form of spores in the nutrient-deprived environment, which may favor temperate phages over strictly lytic phages.

Phages with the ability to lyse different strains of *C. difficile* are summarized in Table 4, along with phage derivatives, such as phage tail-like particles or phage endolysins.

**Table 4.** Phage-based therapies with the ability to cause lysis of *C. difficile*..

Agent	Target	Stage of development	Benefits	Limitations	Reference
ΦCD140	*C. difficile*	Preclinical (hamsters)	Specific lytic activity	Occurrence of phage-resistant clones	^105^
ΦCD27	*C. difficile* NCTC11204 ribotype 001	Preclinical (multi-vessel gut model)	Strong lytic activity; reduction of toxin production	Occurrence of phage-resistant clones	^107^
Four phage cocktail CDHM1, 2, 5, and 6	Collection of 80 *C. difficile* strains	Preclinical (*in vitro* assay, hamsters, wax moth larvae)	Reduced risk of the emergence of resistance; more effective in preventing biofilm formation; no negative effect on commensals; increase in specific commensals	Occurrence of resistance to multiple phages from the cocktail	^108,109^
Broad host phage ΦCD1801	Collection of 15 strains of *C. difficile* ribotype 078	Preclinical (*in vitro* assay)	Expanded lysis spectrum; reduced risk of the emergence of resistance	Less specific	^110^
Engineered phage with redirected CRISPR-Cas3	*C. difficile*	Preclinical (mice)	Improved lysogenic activity; eligible for intellectual property protection	Occurrence and regrowth of phage-resistant clones	^111^
Phage tail-like particles	Collection of 16 strains of *C. difficile* ribotype 027 and 40 other bacterial isolates from 23 different ribotypes	Preclinical (*in vitro* assay)	Absence of viral genetic material or antibiotic resistance genes; killing is highly specific and efficient	Optimization of pharmacokinetic properties of oral formulation is needed	^112,113^
PlyCD_1–174_	Collection of *C. difficile*, including ribotypes 087, 001 and 017	Preclinical (mice)	Broader lysis spectrum; no risk of resistance; no negative effect on commensals	Intrarectal delivery	^114^
Endolysins seq_1, seq_5 and seq_10	Collection of *C. difficile*	Preclinical (mice)	Protection from CDI	Intrarectal delivery	^115^
CD27L	Collection of 30 strains of *C. difficile*, including strain 027	Preclinical (*in vitro* assay)	Broader lysis spectrum; no risk of resistance; no negative effect on commensals; wide pH range stability	Challenging oral delivery, potential short term increase in symptoms due to toxin release	^116^
ΦC2 lysin-human defensin HD_5_ fusion protein	Collection of *C. difficile* strains, including 027, 078, 012 and 087	Preclinical (mice)	Broader lysis spectrum; concomitant lysis and TcdB neutralization	Challenging oral delivery	^117^

### 6.1. Single phage therapy against C. difficile

**Figure 3.**
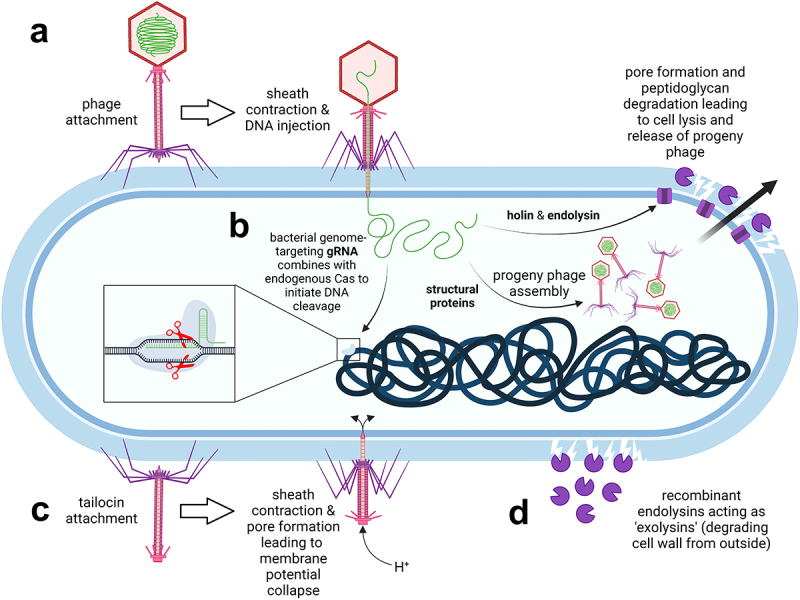
Phage-based therapies against *C. difficile* infections. a. Conventional phage therapy: phages specifically dock to bacterial surface receptors (not shown) via tail fibers and inject their genomic DNA into host bacteria. Progeny phages are released upon bacterial wall disruption by phage-encoded endolysins which gain access to peptidoglycan through holin pores. b. Phages can be exploited as vehicles to deliver guide RNA (gRNA) which combines with the endogenous Cas nuclease of *C. difficile* to catalyze cleavage of the bacterial chromosome. c. Tailocins (phage tail-like particles) have no capsid head and therefore no phage genetic material. Nevertheless, they still selectively attach to bacterial surface receptors and puncture the cell, thereby disrupting the membrane potential. d. Recombinant phage endolysins degrade the peptidoglycan wall from the outside. The image was created with BioRender.

The first study that evaluated the efficacy of phage therapy (Figure 3a) against CDI was conducted with phage ΦCD140 in a hamster model of clindamycin-induced CDI.^105^ The study showed that oral treatment with a single suspension of phages (10^8^ plaque-forming units (PFU)) was able to protect hamsters from *C. difficile*. The majority (>80%) of hamsters treated with phages survived, whereas all control animals died within 96 h of the *C. difficile* challenge. It is worth noting that neutralization of gastric acid with bicarbonate buffer was required for phage survival in the hamster’s gastrointestinal tract (GIT). The phages were recovered from the animal intestine 24 h after administration and were capable of lysing *C. difficile* isolates *in vitro*. However, when the hamsters were re-challenged with *C. difficile* 2 weeks later, the phages failed to prevent re-infection and the hamsters succumbed to the disease due to the development of *C. difficile* lysogens that were resistant to phages.

In more recent studies, Meader et al. demonstrated the specific activity of phage ΦCD27 against *C. difficile in vitro*. In a batch fermentation model,^118^ phage ΦCD27 caused a significant reduction in the bacterial load of vegetative *C. difficile* cells (strain NCTC11204, ribotype 001) after 48 h of exposure without negatively affecting nonpathogenic bacteria. The effect was dose-dependent (improved efficacy was achieved by increasing the phage infection dose from 7 to 10 multiplicity of infection). In a multi-vessel gut model,^107^ phage ΦCD27 reduced the counts of vegetative *C. difficile* cells during a 35-d exposure. In addition to the bacteriolytic activity, phage ΦCD27 reduced the production of *C. difficile* toxins in both models. The prophylactic regimen was more effective in eliminating *C. difficile* cells than the curative regimen, with ~1 log colony-forming units (CFU)/mL of *C. difficile* detected when phages were added before the bacteria, and up to 8 log CFU/mL when phages were added after inoculation of bacteria. The observed regrowth was a consequence of *C. difficile* resistance to phages due to early lysogeny, highlighting the therapeutic limitations of lysogenic phages, which are discussed in more detail below.

### 6.2. Multi-phage therapy against C. difficile

The development of *C. difficile* lysogens resistant to phages has been reported in several studies in which a single *C. difficile* phage was used for therapy of CDI.^105,107,118^ Nale et al.^108^ overcame this issue by using a phage cocktail of four well-characterized, temperate *C. difficile* phages that exhibit lytic activity but also encode integrase genes. In the study, they determined the bactericidal activity of seven phages (10^8^ PFU/mL) against 80 *C. difficile* strains belonging to 21 major epidemic and clinically relevant ribotypes. The phages lysed 78% of the strains and 86% of the ribotypes tested. However, after the initial reduction in bacterial load, treatment with single phages led to the development of phage-resistant colonies. In contrast, an optimized combination of four phages (CDHM1, 2, 5, and 6) caused complete lysis of *C. difficile in vitro* (without the emergence of resistant clones) and delayed the onset of symptoms in a hamster model by reducing *C. difficile* colonization.^108^ The phage cocktail eradicated *C. difficile*, presumably through a complementary effect in which resistant clones of *C. difficile* generated by one phage were susceptible to infection by another phage in the mixture. This cocktail was further tested in the wax moth larvae model of CDI, where administration of single or multiple doses reduced *C. difficile* colonization, resulting in disease prevention during prophylactic treatment and increased larval survival during remedial treatment (60% of larvae survived).^119^ It is worth noting that the best effect was observed when vancomycin was administered before phages. This suggests that phages could potentially be used as an adjunct to vancomycin to prevent disease relapse. As reported in several other studies,^108,109,120^ the experiments on wax moth larvae also showed greater efficacy of phages in prophylactic treatment schedule compared to curative treatment, the latter requiring multiple doses to achieve comparable results. When phages were administered prior to infection with *C. difficile*, more effective bacteriolysis was attained, presumably due to their better distribution in the GIT and temperature acclimation, which seems to be necessary for their activity.^120^ Furthermore, the cocktail of four phages (CDHM1, 2, 5, and 6) also prevented the formation of *C. difficile* biofilms and was able to disrupt established biofilms *in vitro*, albeit to a lesser extent than the pretreated cultures.^119^ Microscopic data showed that the phages penetrated the biofilms by degrading the extracellular polymeric matrix in a biofilm. This activity is attributed to phage-encoded enzymes that are species-specific. The effect of *C. difficile* phages on the gut microbiome was investigated in a batch fermentation model using fecal samples from healthy individuals.^109^ To ensure broad microbial diversity, donors of different ages and ethnic groups were included. The mixture contained ~10^6^ CFU/mL anaerobes and enterobacteria in combination with ~10^5^ CFU/mL enterococci, lactobacilli, and bifidobacteria. The four-phage cocktail (containing CDHM1, 2, 5, and 6) was effective against *C. difficile* (clinical strain CD105LC2, ribotype 014/020), causing a ~ 6-log reduction in bacterial load after 5 h of exposure during the prophylactic regimen, and complete eradication of *C. difficile* after 24 h during both prophylactic and curative treatment. No adverse effects were observed on any of the five bacterial groups of the indigenous human gut microbiome, but rather an increase of approximately 2 logs in the number of lactobacilli, enterobacteria, and total anaerobes in the phage-treated vessel compared to the other treatments. These data suggest that the application of specific phage combinations will be required for optimal therapeutic efficacy of phage therapy against CDI.

### 6.3. Limitations of using temperate phages for therapy and possible solutions

The above studies have demonstrated that the use of phages for the treatment of CDI is feasible. However, because clinical research is lacking, the potential long-term adverse effects are not yet known. Phages could be a solution for preventing the recurrence of CDI, but many hurdles still stand in the way of their clinical development. The major safety concern associated with the therapeutic use of phages is their ability to transfer DNA from one bacterium to another via transduction. During a lytic cycle, generalized transduction can occur, in which a phage encloses a bacterial gene in a virion particle during its assembly and transfers it to another bacterium. The gene may be from anywhere in the bacterial genome, including plasmid DNA. Usually, only a portion of the transferred segment is incorporated into the genome of the recipient bacterium. Apart from generalized transduction, specialized transduction can occur during a lysogenic cycle, in which the bacterial gene is excised from the adjacent genome segment along with the prophage and then inserted into the genome of the next infected bacterium. Through transduction, phages can introduce new virulence factors or antibiotic resistance genes into pathogenic bacteria or convert a non-virulent strain into a virulent variant. A well-known example is the phage-mediated transfer of Shiga toxin to nonpathogenic *E. coli*.^121^ Recently, phage transduction of antibiotic resistance to *C. difficile* has been reported. In a study by Goh et al.,^122^ the phage ΦC2 transferred the erythromycin resistance gene from a donor to a recipient *C. difficile* strain. It has also been found that the introduction of phage genetic elements could affect *C. difficile* toxin production. Following phage infection, increased or decreased toxin production was observed depending on the *C. difficile* strain, phage type, and their specific interaction. The lysogenic phage ΦCD38–2 was reported to cause increased toxin production in an epidemic *C. difficile* strain NAP1/027.^123^ Conversely, in the study by Revathi et al.,^124^ *in vivo* lysogenization of phage ΦCD119 resulted in reduced toxin production in *C. difficile* isolates.

One of the strategies used today to overcome the drawbacks of temperate phages in CDI therapy is phage engineering. Several different approaches are being pursued, including the elimination of genes involved in lysogeny, expansion of host range, extension of half-life, improvement of biofilm disruption activity or enhancement of antimicrobial activity.^125^ The bactericidal activity of temperate phages can be enhanced by the incorporation of a secondary therapeutic payload (Figure 3b). Based on this approach, Selle et al.^111^ developed phage ΦCD24–2, which encodes a bacterial genome-targeting CRISPR (clustered regularly interspaced short palindromic repeat) derived from the sequence of the *C. difficile* 630 CR11 array to achieve sequence-specific killing of *C. difficile*. A recombinant CRISPR-enhanced phage demonstrated a stronger killing ability than its wild-type counterpart both *in vitro* and in a mouse model of CDI. Subsequently, the *cI* repressor and integrase genes were deleted from the *C. difficile* phage ΦCD24–2 genome to prevent the formation of lysogens. In the future, technological advances in phage therapy could increase the chances of patentability and generate revenue to justify development costs. For example, phages engineered using the CRISPR/Cas9 gene editing tool are more likely to receive a patent for unique preparation. Apart from intellectual property protection, phages require their own regulatory framework with adequate production and quality control, which is currently being established.^126^

Another obstacle to the clinical development of phage therapy is the development of bacterial resistance to phages. The constant evolutionary competition between phages and their host bacteria has resulted in bacteria developing numerous defense mechanisms against phage infection. Apart from downregulating phage receptors, bacteria use CRISPR/Cas or type I and type II active restriction-modification systems to destroy phage DNA that has entered the cell.^106^ Furthermore, after incorporation into the bacterial genome, temperate phage can prevent infection of bacterial cells with related phages by occupying the insertion site of a future viral genome. The development of bacterial resistance to phages is widespread. However, phages have co-evolved with their bacterial hosts and have developed various strategies to maintain infectivity.

As demonstrated in the above studies, the cocktail of phages that adhere to different receptors has been successful in overcoming resistance, as bacteria are less likely to become resistant to multiple phages simultaneously. However, interactions between phages in a cocktail are complex and can sometimes be unfavorable. Also, bacteria can develop cross-resistance to multiple phages by forming a capsule that prevents phage binding or by shedding their cell wall in response to environmental stressors.^127^ For these reasons, phage cocktails should be rationally designed and tested to ensure that the different phages in a cocktail do not reduce efficacy by competing with each other. Phages with broad host activity can be used to create an optimal phage cocktail. Recently, Whittle et al. isolated the first phage with broad host activity against *C. difficile* (designated ΦCD1801), which is active against *C. difficile* ribotype 078 and infects 15 of 16 isolates tested.^110^ By using this phage in the binding assay, S-layer protein A was identified as a bacterial cell surface receptor for *C. difficile* phages. The identification of phage receptors allows the search for phages that are active against a broad spectrum of *C. difficile* strains, such as those against specific S-layer cassette types.^110^

In addition to the use of phage cocktails, future approaches to overcome phage resistance in *C. difficile* may involve the use of phages which select for bacteria that suffer genetic trade-offs during phage infection. Genetic trade-offs often occur in biological systems when organisms evolve one trait at the expense of lower performance in another trait. For example, in studies with *Pseudomonas aeruginosa*, it was observed that a phage that binds to an antibiotic efflux pump can downregulate its expression, thus resensitizing the bacteria to antibiotics that were previously excreted from the cell.^103^ Similarly, phages that attach to pili impede bacterial invasion of epithelial cells. Infection with phages that use lipopolysaccharide as a receptor can result in bacterial mutants with reduced fitness and virulence. Phage therapy could benefit from the use of such phages, as this approach is effective when the phages lyse the target bacteria, but also when the bacteria are rendered less virulent or more sensitive to antibiotics after acquiring phage resistance. This avenue could be pursued in the future to improve the therapeutic utility of temperate phages.

### 6.4. Phage tail-like particles

Phage tail-like particles (tailocins; Figure 3c) represent an attractive alternative to phage therapy. They are produced by some strains of *C. difficile* to exert bactericidal activity against competing *C. difficile* strains. These particles belong to the R-type bacteriocins and have a virus-like structure with a contractile tail, nanotube core, base plate, and tail fibers, but no capsid and thus no viral genetic material. The killing specificity of bacteriocins is determined by a receptor binding protein located at the fiber tips. The killing is highly specific and effective. It is initiated by a sheath contraction that projects a nanotube core through the bacterial cell envelope, thus creating a small pore that disrupts the cell membrane potential. Because these particles do not contain genetic material, they cannot be used as gene transfer agents, which would provide a safety advantage over conventional phage therapy or FMT. This makes them suitable as potential prophylactic agents for CDI that can be used to decolonize asymptomatic carriers.^112^ Prevention of *C. difficile* colonization or swift decolonization of a carrier is of utmost importance, especially given the recent spread of epidemic *C. difficile* strain BI/NAP1/027 in health care facilities. Control of person-to-person transmission and elimination of this strain from hospitals has proven difficult because the spores are insensitive to most interventions. To stop the spread of infection, *C. difficile* colonization must be prevented or the carrier decolonized promptly before the pathogen releases spores into the environment. Phage tail-like particles genetically engineered to increase their stability during transit through the GIT were able to prevent colonization of mice exposed to *C. difficile* BI/NAP1/027 spores without detectable alteration of the resident gut microbiota.^113^ The modified tailocins administered orally in a sodium bicarbonate solution (as a buffer against gastric acid) retained their bactericidal activity following transit through the mouse GIT. In the *in vitro* assay, the modified tailocins lysed 16 strains of *C. difficile* belonging to ribotype 027, including the hypervirulent *C. difficile* BI/NAP1/027. In addition to these strains, 40 other bacterial isolates belonging to 23 different ribotypes were susceptible to the modified phage tail-like particles, including ribotypes 001, 015, 046, and the highly toxigenic ribotype 087.

### 6.5. Phage endolysins

Endolysins are peptidoglycan hydrolases used by bacteriophages to lyse host cells in the final step of the bacteriophage lytic cycle. Endolysins pass the membrane through the pores formed by holin and hydrolyze the peptidoglycan layer within, leading to bacterial lysis.^128^ While the peptidoglycan cell wall of Gram-negative bacteria is protected by an additional outer phospholipid layer, the cell wall of Gram-positive bacteria is also accessible from the outside, allowing endolysins to act as “exolysins” and lyse Gram-positive cells (Figure 3d). This can be exploited in the development of so-called “enzybiotics” that could be used instead of antibiotics or phages to treat infections, including those caused by *C. difficile*.^129^ Compared with phages, endolysins are also highly specific to the genus or species, but have a somewhat broader antimicrobial range.^130^ They are efficient in killing bacteria and do not cause the development of bacterial resistance.^131^

Endolysins derived from phages infecting *C. difficile* usually consist of an N-terminal enzymatically active domain (EAD) and a C-terminal cell wall-binding domain (CBD). The role of CBD in antibacterial treatment is controversial. On the one hand, CBD may allow localization of the substrate (peptidoglycan) in the proximity of the catalytic site of the enzyme^132^; on the other hand, it may anchor endolysins to peptidoglycan of post-lytic cell remnants, thus limiting their availability for other cells.^133^ Accordingly, some recombinant endolysins have higher lytic activity when they lack CBD, whereas others have lower or no lytic activity.^129,134^ Also, some studies indicate that EAD alone determines endolysin specificity,^135^ which is somewhat broader compared with endolysins containing CBD.^114,132^ Thus, recombinantly produced EAD may represent a better treatment strategy for *C. difficile* compared with full-length endolysins.

Several endolysins that target *C. difficile*, including PlyCD, CD27L, CDG, and CD11, have been recombinantly expressed and studied *in vitro* and *in vivo*. Endolysin PlyCD consists of 262 amino acids and is active against the super-virulent strain *C. difficile* 630. Compared with full-length PlyCD, EAD of PlyCD (PlyCD_1–174_) was significantly more effective (more than 4 log units) in lysing *C. difficile* while retaining selectivity. In an *in vivo* model, intrarectal administration of PlyCD_1–174_ of mice infected with *C. difficile* spores resulted in an increase in survival from 20% to 45% at day 7, but failed to conclusively demonstrate an overall increase in survival of mice, probably due to problems with administration and distribution. However, PlyCD_1–174_ reduced *C. difficile* colonization by more than 2 log units in an *ex vivo* mouse model.^114^ Endolysins identified in *C. difficile* prophage sequences obtained from metagenome data of 101 healthy individuals were recombinantly expressed and demonstrated lytic activity *in vitro*. Three endolysins (seq_1, seq_5, and seq_10) were administered rectally in an *in vivo* mouse model of *C. difficile* challenge, and increased 7-d survival from 30% (control) to 90% (seq_5 and seq_10) and 100% (seq_1), respectively.^115^ CD27L is derived from bacteriophage φCD27 and is composed of 270 amino acids. It is efficient against approximately 30 strains of *C. difficile*, including the hypervirulent strain 027, and has amidase-3 catalytic activity in EAD,^116^ but has not been tested *in vivo* yet. CD11 and CDG were identified *in silico* based on a sequence homology search of a database of *C. difficile* genomes and expressed recombinantly. They are selective against *C. difficile* and act against a wide range of clinically relevant strains, including strain 630, with a reduction in the number of viable bacteria by more than 4 logs.^136^

The lytic activity of endolysins or their modules can be enhanced by fusing them with other proteins.^137^ An example includes lysin-human defensin fusion protein, which consists of EAD from the bacteriophage ΦC2 and a functional domain from human α-defensin-5. The fusion protein had lytic activity against several *C. difficile* strains, including 027, 078, 012, and 087. Its *in vivo* administration in the CDI mouse model increased survival (from 60% to 100%), decreased the percentage of animals with diarrhea, and reduced intestinal concentrations of *C. difficile* spores and toxins.^117^

## 7. Interfering with immune signaling by delivery or neutralization of cytokines and chemokines

As our understanding of the immunological processes involved in CDI increases, innovative experimental treatments are being proposed as a result of translational research. The intestinal mucosa is no longer considered solely as a physical barrier that prevents microbes from entering the organism and causing systemic infection. Rather, various epithelial cells are increasingly recognized as active components of the immune response, performing tasks such as antigen sampling and secretion of antimicrobial peptides and cytokines.^138^ The following examples of CDI treatment focus on affecting cytokine signaling, achieved either by providing selected cytokines or by neutralizing them (Table 5).

**Table 5.** Cytokines and anti-cytokine antibodies that facilitate clearance of *C. difficile* by modulating the immune system.

Agent	Target	Stage of development	Reference
IL-27	IL-27 receptor	Preclinical (mice)	^139^
Anti-IL-22 and anti-CD160 antibodies	IL-22, CD160	Preclinical (mice)	^140^
IL-33	IL-33 receptor	Preclinical (mice)	^141^
Anti MIP-1α antibody	MIP-1α	Preclinical (mice)	^142^
Anti IL-23 antibody	IL-23	Preclinical (mice)	^143^

Interleukin-27 (IL-27) was shown to significantly induce the expression of cathelicidin LL-37 in primary human colonic epithelial cells *in vitro*.^139^ Furthermore, production of the murine LL-37 orthologue (CRAMP) was impaired in IL-27 receptor-deficient mice after CDI, whereas treatment of wild-type animals with IL-27 enhanced expression of CRAMP in colonic tissue. CRAMP supplementation decreased *C. difficile* bacterial burden in cecal contents and increased survival after *C. difficile* challenge in IL-27 receptor knock-out mice compared with untreated counterparts (survival rates of 70% versus 40% 2 weeks post infection, *p* < .001). Importantly, the IL-27/LL-37 axis was found to be clinically relevant in *C. difficile*-infected patients, as a positive correlation between IL-27 and LL-37 was detected in both serum and stool. Thus, activation of the IL-27/LL-37 axis was proposed as a potential therapeutic strategy for CDI. However, sub-inhibitory concentrations of LL-37 have been shown to promote glycine catabolism in *C. difficile* via induction of glycine reductase genes.^144^ In turn, glycine fermentation stimulates bacterial growth, toxin formation, and sporulation,^145^ indicating an intricate interplay between the host immune response and the pathogen.

Using a mouse model, Sadighi Akha et al.^140^ demonstrated that IL-22 and CD160, a protein anchored to the surface of intestinal intraepithelial lymphocytes, are involved in the phosphorylation of signal transducer and activator of transcription 3 (STAT3) in the context of CDI. Genes encoding antimicrobial peptides, pro-inflammatory chemokines and cytokines were induced to a significantly lesser extent in CDI mice treated simultaneously with anti-IL-22 and anti-CD160 antibodies compared with untreated *C. difficile*-infected animals. This resulted in reduced colonic recruitment of neutrophils in CDI mice treated with anti-IL-22/anti-CD160, and thus an anti-inflammatory effect attributable to limited host-mediated damage. However, anti-IL-22/anti-CD160 co-therapy (or any other immunosuppressive immunotherapy) during CDI could ultimately induce an opposite effect, as excessive dampening of mucosal immune mechanisms could actually promote epithelial damage and *C. difficile* colonization.

Nagao-Kitamoto et al.^146^ recently demonstrated that IL-22, which is induced by colonization of the gut microbiota, plays a critical role in the prevention of CDI by regulating the glycosylation of host N-linked glycans. In turn, IL-22-modulated glycosylation promotes the growth of commensal bacteria (e.g., the succinate-consuming *Phascolactobacterium* spp.) that compete with *C. difficile* for the nutritional niche. By comparing the transcriptome of the entire cecal tissue of mice infected with the highly virulent CDT toxin-expressing *C. difficile* ribotype 027 strain R20291 or its attenuated isogenic mutant (lacking the CDTb receptor-binding region) with uninfected controls, Frisbee at al.^141^ also observed that IL-33 is upregulated in response to increasing severity of CDI. Treatment with IL-33 protected mice from *C. difficile*-associated disease, reduced mortality (30% versus 70% in the untreated control group), and prevented epithelial barrier disruption during infection. IL-33 activates type-2 innate lymphoid cells, and this mechanism is critical for defense against *C. difficile* associated colitis in both mice and humans.

Wang et al.^142^ analyzed the profile of cytokines produced by mouse and human colonic explants after TcdA and TcdB exposure. They found that TcdA induces the expression of macrophage inflammatory protein 1α (MIP-1α), promoting immune cell infiltration and inflammatory responses. Concomitantly, the chloride/bicarbonate exchanger protein SLC26A3 (colonic solute carrier family 26, member 3 involved in fluid homeostasis) was downregulated. This situation was similar to that observed in CDI patients. Downregulation of SLC26A3 is associated with damage to the epithelial layer, which allows the toxin easy access to the mucosa. A MIP-1α-neutralizing antibody prevented the death of *C. difficile*-infected mice when injected intraperitoneally (survival rates of 100% versus 80% in untreated controls, *p* < .001). Neutralization of MIP-1α reduced colon damage, inhibited the production of IL-1β, and restored the expression of SLC26A3 in the colon of mice and prevented relapse of vancomycin-associated disease. Moreover, the knock-down of SLC26A3 *in vivo* increased mortality in a recurrent CDI mouse model, whereas its restoration reduced CDI relapse (although it did not affect survival). These results argue for anti-MIP-1α therapy in CDI.

Aberrant adaptive responses can also lead to severe CDI outcomes.^147^ Saleh et al.^148^ have investigated the mechanism by which prior colitis exacerbates CDI. Dextran sulfate sodium (DSS) was used to induce colitis in mice, and the animals were infected with *C. difficile* after a 2-week recovery period. They noted that the severity of CDI depended on CD4+ T lymphocytes that were still present after the colitis-associated inflammation subsided. Moreover, adoptive transfer of T helper (Th) 17 cells to naive mice increased CDI-associated mortality by increasing IL-17 production. Blocking IL-17 signaling protected DSS mice from severe disease at early stages of infection, but was ineffective at later stages of infection. Importantly, in CDI patients, serum levels of IL-6 and IL-23 (cytokines upstream of pathogenic Th17 cells) correlate with CDI severity.^143,148^ Patients with the highest serum levels of IL-6 (first quartile) were 7.6 times more likely to die from infection than those in the lowest quartile (*p* = .0009). Therefore, it was hypothesized that targeting Th17 cells (or their effector cytokines) may protect against severe CDI in patients with inflammatory bowel disease.^148^ In line with that, IL-23a (p19) knock-out mice were fully protected from *C. difficile* challenge and had lower morbidity, whereas their wild-type counterparts had significantly lower survival (16.7% on day 2 of infection).^143^ Similar results were obtained by neutralizing IL-23 using a monoclonal antibody.

## 8. Non-antibiotic small molecule agents against C. difficile

Most drugs against *C. difficile* belong to the group of antibiotics, which are small-molecule agents. However, in recent years, attention is shifting to small-molecule agents without direct bacteriolytic or bacteriostatic activity that cause extensive perturbations of intestinal microbiota. Currently, a major effort is underway to repurpose drugs by screening libraries of drugs approved for unrelated diseases for their potential activity against *C. difficile*. Such drugs have the advantage of confirmed safety. Non-antibiotic small-molecule agents against *C. difficile* inactivate toxins, activate immune signaling pathways, bile acid synthesis, or have other mechanisms of action (Table 6).

**Table 6.** Small-molecule agents against *C. difficile* that prevent the activity of *C. difficile* toxins, activate the immune system, affect bile acid synthesis, or exert their effects via other mechanisms.

Agent	Target	Stage of development	Reference
VB-82252	Inhibition of UDP-glucose hydrolytic activity of TcdA and TcdB	Preclinical (mice)	^149^
Ebselen	Inhibition of toxin cysteine protease domain	Preclinical (mice)	^150^
Inositol hexakisphosphate	Toxin TcdB auto-proteolysis induction	Preclinical (mice)	^151^
Niclosamide	Increase of pH of endosomes, prevention of toxin entry	Preclinical (mice)	^152^
Indole-3-carbinol	Activation of aryl hydrocarbon receptor	Preclinical (mice)	^153^
SR1001	Inhibition of (ROR)γt	Preclinical (mice)	^154^
Amoxapine, doxapram, trifluoperazine	Induction of chemokines, IL-33 and IL-22	Preclinical (mice)	^155^
MRS2578	Inhibition of P2Y6 receptor	Preclinical (mice)	^156^
CamSA	Inhibition of spore germination	Preclinical (mice, hamsters)	^157–159^
Obeticholic acid	Inhibition of bile acid synthesis	Preclinical (mice)	^160^
Auranofin	Inhibition of thioredoxin reductase	Preclinical (mice)	^161,162^
Misoprostol	PGE1 analog	Preclinical (mice)	^163^
5FDQD	Riboflavin analog, riboswitch activation	Preclinical (mice)	^164^

Inactivation of toxin activity and consequent reduction in severity of CDI can be achieved in different ways, as will be described below. The clostridial toxins TcdA and TcdB are glucosyltransferases that exert their toxic effects by inhibiting host Rho GTPases. VB-82252 is a highly potent small-molecule inhibitor of the UDP-glucose hydrolysis activity of TcdA and TcdB that protects cells from intoxication with either of the two toxins.^149^ Oral administration of VB-82252 prevented inflammation in a murine intrarectal toxin challenge model and reduced weight loss and intestinal inflammation during acute disease in recurrent CDI model. Ebselen, a low molecular weight organoselenium compound currently in clinical trials for a clinically unrelated indication, was identified by targeted screening with an activity-based probe for the protease domain (Figure 2b) and shown to be a potent inhibitor of the toxins’ cysteine protease domain (IC_50_ = 6.9 nM), preventing hexakisphosphate-induced release of the toxic glucosyltransferase domain.^150^ Mice injected with TcdB and pretreated with ebselen (100 mg/kg by oral gavage) for 5 d had a survival rate of 100% compared to the control group, in which 60% of mice died after 1 d and 100% after 2 d. Animals receiving ebselen also had less infiltration of inflammatory cells in the cecum and proximal colon and showed less submucosal edema and mucosal hypertrophy compared to the control group. Inositol hexakisphosphate (IP6) induces auto-proteolysis of the toxin TcdB in the intestinal lumen prior to the cellular uptake, thus preventing its toxic effect.^151^ Oral administration of IP6 analogues (in which phosphates were substituted with sulfate and/or thiophosphate groups to evade calcium chelation) attenuated inflammation and promoted survival in mouse models of CDI. At the same time, they were well tolerated and showed no signs of toxicity or weight loss in mice at a dose of 2 mg/kg. An alternative mechanism for inhibiting toxin entry was found in the study by Tam et al.^152^ Niclosamide is an antihelmintic drug that was shown to inhibit TcdB toxin activity in a cell model screening. Niclosamide slightly increases the pH of endosomes, thereby preventing the toxin from entering into the cytosol of colonocytes. Niclosamide (or its more water-soluble ethanolamine salt) had a protective effect on primary and recurrent CDI in mice administered with *C. difficile* spores. It had no effect on *C. difficile* growth *in vitro*, and did not affect the structure or composition of the microbiota. Recently, amiodarone, an antiarrhythmic drug blocking potassium, sodium, and calcium channels, was shown to be a potent inhibitor of TcdA and TcdB exotoxins.^165^ In an elegant study, the authors demonstrated that amiodarone prevents glucosyltransferase domain release into the cytosol by insertion into and blockage of the toxins’ translocation pore. *In vitro*, the drug was active against the toxin variants from the clinically relevant epidemic *C. difficile* strain NAP1/027 at concentrations comparable to those reached in the plasma of patients treated for arrhythmia.

The immune system plays a critical role in containing CDI, and interfering with immune pathways is a viable approach to combat *C. difficile*. Indole-3-carbinol (I3C) is a safe dietary supplement normally found in foods as a metabolite of glucobrassicin and has been tested in mice.^153^ I3C acts as a weak aryl hydrocarbon receptor (AHR) ligand and represents a potential new therapy for CDI. Administration of I3C to mice resulted in an increase in regulatory T cells, type 3 innate lymphoid cells, and intestinal T cells in mice. A significant increase in survival (67%) was observed in these mice after infection with *C. difficile* spores compared to control mice treated with antibiotics (20%). The transcription factor (ROR)γt (retinoic acid-receptor-related orphan receptor) is characteristic for Th17 cells.^166^ Wang et al.^154^ developed SR1001, a small molecule inverse agonist of (ROR)γt that inhibits the expression of genes preferentially expressed in Th17 cells, such as those encoding IL-6, IL-17A, and TNF. Treatment with SR1001 improved disease status in mouse models of recurrent CDI. Furthermore, the same group showed that mice regularly fed with butyrate were protected from dextran sulfate sodium and *C. difficile*-induced colitis.^167^ Butyrate effectively suppressed the expression of proinflammatory cytokines and activated SIRT1 histone deacetylase/mTOR axis to impede Th17 cell differentiation. Notably, mice receiving a selective SIRT1 inhibitor with butyrate treatment displayed marked colitis, characterized by high Th17 cell levels and Th17-associated cytokine profile, providing evidence that butyrate therapeutic effect is SIRT1-dependent.

In another attempt to repurpose approved drugs for CDI, the antidepressant amoxapine, the breathing stimulant doxapram, and the antipsychotic trifluoperazine were found to protect against CDI. These drugs increased the expression of genes encoding cytokines IL-33 and IL-22, which are involved in the recruitment of neutrophils, cells that are critical for clearing CDI and are associated with increased mortality in their absence. Amoxapine also caused downregulation of several genes known to adversely affect CDI, such as those encoding IL-1ß, IL-6, IL-23, and TNF. All three drugs provided significant protection against lethal infection in mice, with amoxapine providing 70% protection, doxapram 50% protection, and trifluoperazine 55% protection, at doses lower than therapeutic doses in humans.^155^ The clostridial toxins TcdA and TcdB induce the release of the neutrophil-attracting chemokine CXCL8 and trigger intestinal barrier dysfunction. This is caused by the action of extracellular UDP released from stressed or dying cells, leading to the activation of the purinergic receptor P2Y6. The P2Y6 receptor is a G protein-coupled receptor involved in the mobilization of intracellular calcium, stimulation of protein kinase C, and induction of Rho-associated kinase signaling that modulates cell–cell contacts and triggers barrier dysfunction. The selective inhibitor of the P2Y6 receptor, MRS2578, attenuated TcdA/B-induced inflammation and intestinal permeability in a model of intrarectal toxin exposure in mice.^156^

Bile acids are known to modulate the activity and virulence of *C. difficile*.^157^ Meta-aminosulphonate cholate derivative (CamSA) is a bile salt analog that has been shown to be a potent competitive inhibitor of taurocholate-mediated *C. difficile* spore germination. Administration of 50 mg/kg CamSA to mice prevented CDI, and no toxicity was observed. Inhibition of spore germination was confirmed in mice feces in which only spores were detected.^158^ In hamsters, CamSA doubled the mean time to death, but was alone insufficient to prevent CDI. When combined with vancomycin, 70% of hamsters survived *C. difficile* challenge and only minor changes in microbiota composition were observed.^159^ Obeticholic acid is an antagonist of the farnesoid X receptor (FXR) and is approved as an orphan drug for the treatment of primary biliary cholangitis. Administration of obeticholic acid to high fat-diet mice resulted in decreased primary bile acid synthesis, reduced numbers of *C. difficile* bacteria, and improved outcome of infection.^160^

Other mechanisms of action against *C. difficile* have also been proposed, including antioxidative enzyme inhibition, enzyme co-factor metabolism, and mucosal protection. Auranofin [2,3,4,6-tetra-*O*-acetyl-1-thio-β-D-glycopyranosato-*S*-(triethyl-phosphine) gold] is an oral gold (I) compound that has been used to treat rheumatoid arthritis and has a well-defined toxicity profile. Auranofin is a potent inhibitor of selenoprotein synthesis, but this is not its antibacterial mechanism of action; rather it has been speculated that auranofin exerts bactericidal activity by inhibiting thioredoxin, a disulfide reductase enzyme responsible for protecting cytosolic components against oxidative stress.^168^ Auranofin decreased *C. difficile* sporulation and toxin production under *in vitro* conditions and in *C. difficile*-infected mice. Although it protected mice from developing the clinical symptoms of CDI (diarrhea and changes in physiological appearance), the infected mice treated with auranofin still lost weight.^161^ In further studies, the authors demonstrated that auranofin protected mice from CDI, with a 100% survival rate at a relatively low dose (0.125 mg/kg) that could also be administered clinically in human patients.^162^ Non-steroidal anti-inflammatory drugs (NSAIDs) have been observed to disrupt the gut microbiota and dramatically exacerbate the severity of CDI in mice. Misoprostol is a synthetic prostaglandin E1 analog approved for the prevention or treatment of gastric and duodenal ulcers in patients taking NSAIDs because of its protective effects on the gastric mucosa. Zackular et al.^163^ have shown that misoprostol protects mice against *C. difficile*-associated mortality, intestinal pathology, and intestinal permeability. Mice treated with misoprostol showed reduced weight loss, less severe diarrhea, and significant recovery of the microbiota. Homeostasis of the essential coenzymes flavin mononucleotide and riboflavin in *C. difficile* is controlled by riboswitches, regulatory segments of mRNAs that regulate the expression of encoded proteins after binding specific metabolites. 5FDQD is a riboflavin analog that binds to these riboswitches and has potent and rapid bactericidal activity.^164^ 5FDQD completely prevented the onset of lethal antibiotic-induced CDI in C57BL/6 mice when administered at a dose of 10 mg/kg twice daily for 5 d. 5FDQD was relatively specific with very low activity against strains of the genera *Bacteroides, Lactobacillus, Bifidobacterium, Actinomyces*, and *Prevotella*.

## 9. Conclusion and future prospects

CDI remains a serious and costly medical problem, especially in hospital settings. Antibiotics are often ineffective, and provide only temporary improvement because they kill vegetative cells, while the spores remain intact, leading to further recurrence of the disease. Moreover, the use of antibiotics is an important factor in triggering the CDI by disrupting the composition of the intestinal microbiota. Therefore, new therapies are in high demand. This review focuses mainly on new or complementary strategies, some of which are in the preclinical development phase, while others have already entered clinical trials or are in clinical use. Despite many promising results, preclinical drugs are inherently limited by the lengthy clinical trial process that lies ahead of them.

Microbial therapies represent a new therapeutic approach for CDI. Moreover, FMT and defined bacterial mixtures are becoming a potential success story, opening further opportunities for microbial therapeutics also in other areas of medicine. As they have been discussed in detail elsewhere, they are not the focus of the present work. Instead, we focus on a wide range of other possibilities that have received far less attention. They include therapies at various stages of development, from clinically approved monoclonal antibodies and antibiotic neutralizers in late-stage clinical trials to therapies thus far evaluated only in animal models. These therapeutics include proteins, viruses, and small molecules. They are mostly administered orally, some also parenterally. Special mention should be made of recombinant microorganisms, which serve as advanced delivery vehicles for genes encoding therapeutic proteins. These include recombinant viruses (adenoviruses),^67,84^ bacteria (lactobacilli),^64^ or yeasts (*Saccharomyces boulardii*)^65^ that produce antibodies or antibody fragments against *C. difficile* toxins *in situ* in the human body. This eliminates the need for repeated administration and provides a cost-effective solution for protein delivery.

Monoclonal and polyclonal antibodies, nanobodies, and affinity proteins developed by *in vitro* molecular evolution have been used to bind and neutralize *C. difficile* toxins. Monoclonal antibodies have become widely accepted as effective biologics, with bezlotoxumab (against TcdB) and actoxumab (against TcdA) leading the way in the treatment of CDI. After a phase III comparison of the two antibodies,^50^ bezlotoxumab was approved for use, whereas actoxumab was abandoned due to lack of efficacy. Other agents have been tested primarily in animal studies, and polyclonal antibodies have shown promising results; however, their use is limited by the fact that they must be administered orally. This problem was addressed by the development of oral formulations containing antacids and protease inhibitors to protect the antibodies from denaturation and proteolysis.^56^ The advantage of nanobodies and binders based on alternative scaffolds is their relatively small size, which facilitates protein engineering and the joining of multiple molecules in tandem fusions, resulting in improved avidity and neutralization ability. Wider use of affinity proteins in general is hampered by mutations in the sequences of toxins in emerging virulent strains that may render existing binders obsolete. Targeting conserved regions may provide a solution. *C. difficile* toxins have also been targeted by antibacterial peptides. Although they can cause direct bacterial lysis, charge-based neutralization of toxins or prevention of apoptosis seems to be the most important mode of action in CDI. To date, studies have been limited to mouse models. Because antimicrobial peptides can be relatively easily grafted onto other proteins, they may represent a possibility for future protein-based combination therapies.

Another therapeutic option is directed to the modulation or preferably the protection of the microbiota. Unlike other agents, this is a preventive rather than a curative approach, achieved by the neutralization of antibiotics in the colon lumen. DAV132 has been tested in a phase III clinical trial, and has the potential to become the next approved drug for CDI.^99^ The advantage of this approach is its relative simplicity and safety, as well as the potential for widespread preventive use when antibiotics are administered in a hospital setting.

Preclinical studies with phages and their derivatives have demonstrated their specific activity against *C. difficile*, confirming the feasibility of this approach for the treatment of CDI. However, studies have also shown a high frequency of lysogeny of *C. difficile* phages that can alter bacterial physiology and must be considered when developing therapies. Advances in sequencing technologies and molecular tools coupled with machine learning^169^ are increasingly being used to overcome the limitations of lysogenic phages. Also, the extreme diversity of phages can be exploited for the discovery of naturally evolved phages with properties suitable for therapy (such as potent antibacterial activity, good safety profile, and the ability to reach target bacteria *in situ*) by generating large, well-characterized, and annotated phage libraries.^103^ The narrow specificity of phages provides the opportunity for effective niche therapies against specific strains or ribotypes. Here, methods that allow rapid identification and characterization of highly efficient phages against a particular bacterial isolate will be critical for the successful development of effective phage therapeutic that may combine multiple strains. In addition, the potential of phage engineering with new possibilities such as phage-like particles without genetic material^112,113^ or phages that deliver specific CRISPR gRNA against *C. difficile* may be exploited for targeted *C. difficile* therapy development.^111^ *C. difficile* endolysins have not yet entered human clinical trials, although they have shown favorable safety and toxicity profiles in preclinical animal studies.^170^ The efficacy of endolysins in mice depended on the route and timing of administration and was more effective with rapid onset of treatment.^171^ The major experimental challenge remains effective delivery in the intestine.^114^ Therefore, it is unlikely that therapy with phages or their derivatives will completely replace antibiotics; instead, it could be used in combination, taking advantage of the strengths of both treatments.

Modulation of the immune response represents another viable strategy for CDI treatment. The present results are all at a relatively early stage of research, and further studies are needed to substantiate the new approach. This strategy will likely remain a complementary option to other treatments, as it is unlikely to result in complete eradication of *C. difficile* and possible adverse effects of immune interference are to be expected. On the other hand, the advantage of this approach is the considerable availability of already developed or approved treatment options, such as monoclonal antibodies or recombinant cytokines, which were originally developed for other diseases.

Small-molecule agents represent a group of heterogeneous compounds with different mechanisms of action that, taken individually, sometimes shed additional light on the mechanisms of *C. difficile* pathology. Their common characteristics are low molecular weight and lack of direct bactericidal activity. Several of these molecules are repurposed, which has the advantage of confirmed safety and may result in a more straightforward approval process and a shorter time to market.

In summary, with advances in genomics, molecular and structural biology, immunology, biotechnology, and high-throughput screening approaches, the antimicrobial toolbox to combat CDI is now more diverse than ever before. Antibiotics remain essential drugs for curing acute CDI, but novel agents with various mechanisms of action hold promise for effective complementary treatment and prevention of recurrent CDI in the future.

## Data Availability

The authors confirm that the data supporting the findings of this study are available within the article.
